# Retrospective comparison of flot and modified dcf as first-line chemotherapy in metastatic gastric adenocarcinoma

**DOI:** 10.55730/1300-0144.5496

**Published:** 2022-07-28

**Authors:** Fatih GÜRLER, Deniz Can GÜVEN, Ergin AYDEMİR, Osman SÜTÇÜOĞLU, Bediz KURT İNCİ, Zafer ARIK, Şuayib YALÇIN, Nuriye ÖZDEMİR, Ahmet ÖZET, Ozan YAZICI

**Affiliations:** 1 Department of Medical Oncology, University of Health Sciences, Dr. Abdurrahman Yurtaslan Oncology Training & Research Hospital, Ankara, Turkey; 2 Department of Medical Oncology, Faculty of Medicine, Hacettepe University, Ankara, Turkey; 3 Department of Internal Medicine, Faculty of Medicine, Gazi University, Ankara, Turkey; 4 Department of Medical Oncology, Faculty of Medicine, Gazi University, Ankara, Turkey; 5 Department of Medical Oncology, Aksaray Training & Research Hospital, Aksaray, Turkey

**Keywords:** FLOT, gastric cancer, gastroeosephageal cancer, modified DCF

## Abstract

**Background/aim:**

The aim of our study was to compare the efficacy and the safety of the FLOT and the modified DCF (mDCF) regimens in patients with metastatic gastric (GC) and gastroesophageal junction (GEJ) adenocarcinoma as first-line treatment.

**Materials and methods:**

The medical records of 72 patients were retrospectively reviewed. Survivals and hematological adverse events of the patients were examined. Factors affecting survivals were analyzed in univariate analysis. A multivariate analysis was performed with the factors contributing to survivals in univariate analysis.

**Results:**

The median PFS (mPFS) was 10.1 months (95% CI, 6.8–13.4) in the FLOT arm (n = 33) and 7.4 months (95% CI, 9.1–21.6) in the mDCF arm (n = 39) (p = 0.041). The median OS (mOS) was 12.9 months (95% CI, 9.7–16.1) in the FLOT arm and 15.4 months (95% CI, 9.1–21.6) in the mDCF arm (p = 0.622). It was found that all grade neutropenia was 51.3% vs. 72.7% (p = 0.063), febrile neutropenia was 8.3% vs. 6.3% (p = 0.743), and thrombocytopenia was 48.7% vs. 51.5% (p = 0.813) in the FLOT and mDCF arms, respectively. Anemia was 59% in the FLOT arm and 100% in the mDCF arm (p < 0.001). Grade 3–4 anemia was 7.7% in the FLOT arm and 24.2% in the mDCF arm (p = 0.052).

**Conclusion:**

It was shown that the mPFS was significantly increased in the FLOT arm compared to the mDCF arm as the first-line treatment in patients with metastatic GC and GEJC. Hematological adverse events were more favorable in the FLOT arm than in the mDCF arm.

## 1. Introduction

Gastric cancer is the fifth most common malignancy worldwide, and is the third leading cause of cancer-related deaths according to GLOBOCAN 2018 [1]. Approximately 31% of the patients at the time of diagnosis are in the metastatic stage, and it was reported that 1-year survival was 22.9% [2]. In metastatic disease, the aim is to prolong the overall survival (OS) and improve the quality of life. Therefore, researching and comparing effective chemotherapy regimens with tolerable side effect profiles is an area of ​​research that attracts the attention of clinicians. Trials of combination regimens have enabled the rapid development of treatment protocols [3–8]. In the phase III TAX-325 trial, it was found that median time to tumor progression (mTTP) (5.6 months vs. 3.7 months; HR 1.47; 95% CI, 1.19–1.82; p < 0.001) and mOS (9.2 months vs. 8.6 months; HR 1.29; 95% CI, 1.00–1.60; p = 0.02) were significantly improved in favor of the DCF arm compared to the CF arm. The objective response rate was 36.7% in the DCF arm. However, grade 3–4 adverse events were 69% in the DCF arm and 59% in the CF arm. Notably, this discordant adverse event profile was also observed to be reflected in compliance with treatment [9]. Many modification trials have been carried out to increase patient tolerance without reducing the effectiveness of the treatment [10–14].

The FLOT regimen proved its efficacy in a perioperative setting with the FLOT4 trial in locally advanced resectable gastric and gastroesophageal adenocarcinoma patients [15]. However, there is a limited number of FLOT studies in patients with metastatic GC. In the three-armed AIO-FLOT3 trial, arm B consisted of limited mGC, and arm C consisted of extensive mGC patients. In the arm B, improved survival was shown in the group that underwent surgery after neoadjuvant FLOT therapy. In the arm C, mPFS was 6.3 months (95% CI, 5.0–7.6), and mOS was 10.7 months (95% CI, 9.1–12.8) [16]. In another phase 2 study with mGC patients, FLOT efficacy was demonstrated. In the study, 54 patients were treated, and 93% of the patients had metastatic disease. The objective response rate (ORR) was 57.7%, median progression-free survival (mPFS) was 5.2 months, and mOS was 11.1 months. Grade 3–4 neutropenia was observed in 26 (48.1%) patients [17]. Although both FLOT and mDCF regimens contain docetaxel, 5-FU and platinum, their adverse event profiles and efficacy might be different. Numerically higher response rate and overall survival, and lower grade 3–4 toxicity rate with the FLOT regimen in the phase II study compared to those of with the DCF regimen in the TAX325 study raised the question of whether the FLOT regimen might be a more tolerable and effective regimen than the DCF regimen.

The purpose of our study was to compare the efficacy and safety of the mDCF and the FLOT regimens as a first-line treatment in patients with metastatic gastric and gastroesophageal junction adenocarcinoma retrospectively.

## 2. Materials and methods

### 2.1. Patients and treatment protocols

In the study, patients who were admitted to departments of medical oncology in Gazi University and Hacettepe University between October 2013–February 2020 and diagnosed with mGC/mGEJC were reviewed. Inclusion criteria were defined as; >18 years of age, de novo or recurrent metastatic disease, availability of all patient records, taking FLOT or mDCF regimens as a first-line treatment in metastatic disease, HER2 negativity (immunohistochemistry-IHC 0, IHC 1+, in situ hybridization-ISH negative). Exclusion criteria were defined as; secondary malignancy, non-adenocarcinoma histological subtypes or HER2 positivity (IHC3+, ISH positive). Seventy-two patients were included in the study. Diagnosis date, treatments received, treatment start and end dates, adverse events follow-ups, complete blood count, and biochemistry analysis were obtained from the patient records. Progression-free survival was defined as the time in months from first-line treatment initiation to progression (CT, PET-CT), intolerable toxicity, or death. OS was defined as the time in months from diagnosis of metastatic disease to death or patient’s last visit.

The mDCF regimen was used as docetaxel 60 mg/m^2^ (1-h intravenous infusion) plus cisplatin 60mg/m^2^ (2-h intravenous infusion) on day 1, followed by 5-fluorouracil 600 mg/m^2^/day (continuous intravenous infusion) for 5 days every 3 weeks in our institutions. The FLOT regimen was used as docetaxel 50 mg/m^2^ (1-h intravenous infusion) plus oxaliplatin 85 mg/m^2^ (2-h intravenous infusion) plus folinic acid 200 mg/m^2^ (2-h intravenous infusion) on day 1 and followed by 5-fluorouracil 2600 mg/m^2^ (24-h intravenous infusion) every 2 weeks.

Ethics committee approval of the study was obtained from Gazi University Review Board (01.12.2020, 2021–28).

### 2.2. Statistical analysis

All statistical analyses were performed using SPSS version 22.0. Descriptive statistical analysis was performed to show the distribution of variables in the population. Non-normally distributed continuous variables were reported using the median (interquartile range), and categorical variables were reported using the Pearson chi-square test. A Kaplan-Meier test was used to generate survival curves, and a log-rank test was used to compare OS and PFS results. A univariate analysis was performed to determine the contribution of age, gender, metastatic regions, number of metastatic sites, primary tumor localization, tumor differentiation, mucinosis, ECOG PS and chemotherapy on patients OS and PFS. A multivariate analysis was performed for variables that had significant contributions to survival in univariate analysis, and for variables that were thought to be clinically significant. All statistical tests were 2-sided (2-sided), and the significance value was accepted as p < 0.05.

## 3. Results

The study included 72 patients diagnosed with recurrent/de novo metastatic GC/GEJC and administered mDCF (n = 33) or FLOT (n = 39) regimens as a first-line treatment. Eleven patients (15.3%) had recurrent metastatic disease, and 61 patients (84.7%) had de novo metastatic disease in the whole cohort. It was observed that the tumor originated from GEJ and cardia in 27.8% (n = 20) of the patients. The most common metastasis sites were peritoneum [51.4% (n = 37)], liver [43.1% (n = 31)] and bone [16.7% (n = 12)], respectively. The clinicopathological characteristics of the patients in the modified DCF and FLOT groups are shown in Table 1.

**Figure 1:**
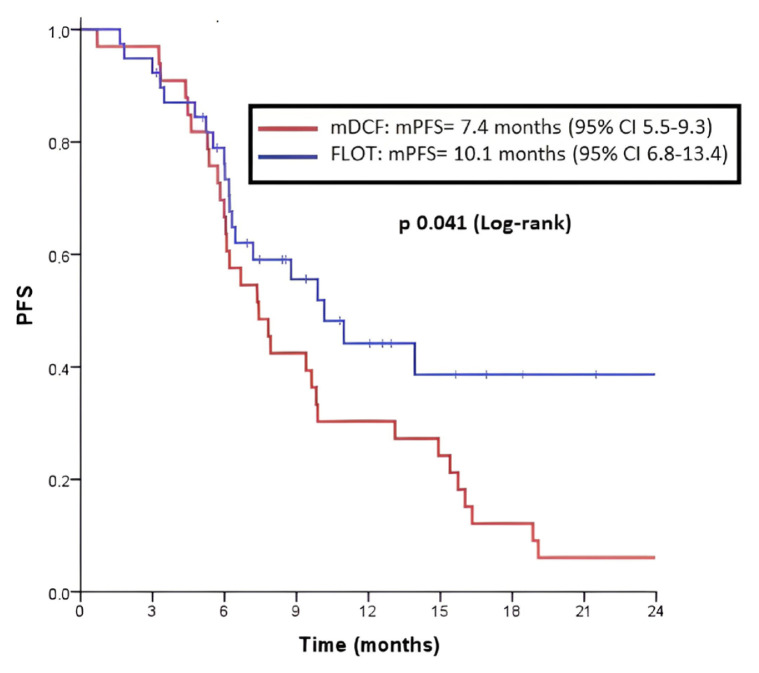
Progression-free survival (PFS) in patients with metastatic GC and GEJC with first-line treatment.

The mPFS was 10.1 months (95% CI, 6.8–13.4) in the FLOT arm and 7.4 months (95% CI, 5.5–9.3) in the mDCF arm, and the difference was statistically significant (p = 0.041) (Figure 1). The analysis of variables that may affect progression-free survival was examined. The mPFS was 6.3 months (95% CI, 5.0–7.5) in the group without liver metastasis, and 13.1 months (95% CI, 7.3–18.8) in the group with liver metastasis (p = 0.029). The mPFS was 13.9 months (95% CI, 8.4–19.3) months in the group without peritoneal metastasis and 6.2 months (95% CI, 5.6–6.7) in the group with peritoneal metastasis (p < 0.001). The mPFS was 13.1 months (95% CI, 5.9–20.2) in the group with number of metastatic sites <2, and 6.6 months (95% CI, 5.3–7.9) in the group with number of metastatic sites ≥2 (p = 0.005). It was found that the mPFS was 9.3 months (95% CI, 7.1–11.6) in the ECOG PS of 0–1 group and 3.3 months (95% CI, 0.6–6.0) in the ECOG PS of 2 group (p = 0.002). A multivariate analysis was performed with variables that had a significantly positive contribution to PFS in the univariate analysis. It was observed that in the multivariate analysis, ECOG PS of 2 (HR 10.38; 95% CI, 2.15–49.9; p = 0.004) and number of metastatic sites ≥2 (HR 1.93; 95% CI, 1.00–3.72; p = 0.048) increased the risk of progression. In the multivariate analysis, the FLOT regimen was devoid of the positive contribution to the risk of progression (HR 0.71; 95% CI, 0.39–128; p = 0.262) (Table 2).

**Figure 2:**
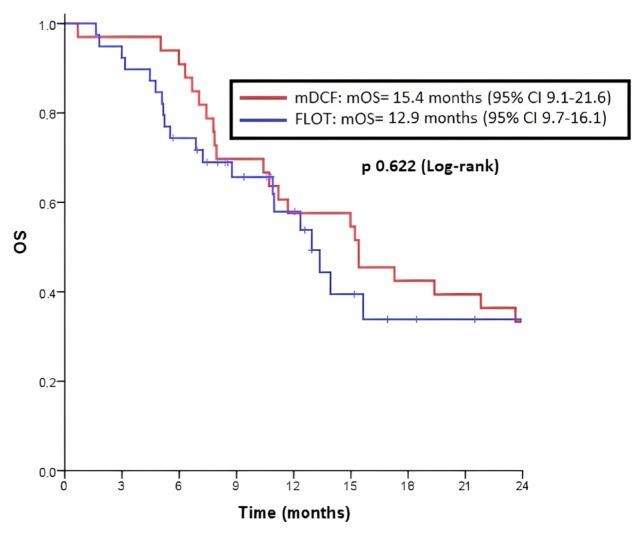
Overall survival (OS) in patients with metastatic GC and GEJC.

The mOS was 15.4 months (95% CI, 9.1–21.6) in the mDCF arm and 12.9 months (95% CI, 9.7–16.1) in the FLOT arm, and the difference was not statistically significant (p = 0.622) (Figure 2). The analysis of variables that may affect the overall survival was examined. It was observed that the mOS was 10.7 months (95% CI, 9.3–12.0) in the group with bone metastasis, 17.2 months (95% CI, 8.7–25.7) in the group without bone metastasis (p = 0.005). The mOS was 25.5 months (95% CI, 8.2–42.9) in the group with number of metastatic sites <2, and 12.3 months (95% CI, 9.2–15.5) in the group with number of metastatic sites ≥2 (p = 0.019). The mOS was 15.2 months (95% CI, 12.5–17.8) in ECOG PS of 0–1 group and 5 months (95% CI, 0–10.6) in ECOG PS of 2 group (p < 0.001). A multivariate analysis was performed with variables that had a significantly positive contribution to OS in the univariate analysis and chemotherapy variable (mDCF or FLOT), which was thought to be clinically significant. It was found that in the multivariate analysis, bone metastasis (HR 2.56; 95% CI, 1.22–5.35; p = 0.012) and ECOG PS of 2 (HR 8.78; 95% CI, 2.44–31.5; p = 0.001) increased the risk of death. However, the FLOT regimen did not reduce the risk of death in the multivariate analysis (HR 1.47; 95% CI, 0.79–2.72; p = 0.219) (Table 3). After the progression under the first-line treatment, 23.3% (n = 7) of the patients in the FLOT arm, and 76.7% (n = 23) in the mDCF arm received second-line chemotherapy (p < 0.001). As a second-line therapy, 77.8% (n = 21) of the patients in the mDCF arm, and 22.2% (n = 6) of the FLOT arm received combined chemotherapy (p < 0.001) (Table 4). In our study, two patients underwent metastasectomy in the mDCF arm, and the overall survivals of these patients were calculated as 39.6 months and 32.6 months.

The disease control rate (DCR) was 84.6% in the FLOT arm and 72.7% in the mDCF arm (p = 0.216). The ORR was 59% in the FLOT arm and 48.5% in the mDCF arm (p = 0.373). The complete response rate was 5.1% vs. 6.1% (p = 0.863), and the partial response rate was 53.8% vs. 42.4% (p = 0.334), in the FLOT arm and mDCF arm, respectively. The progressive disease rate was 15.4% in the FLOT arm, and 27.3% in the mDCF arm (p = 0.216) (Table 5).

It was found that neutropenia was 51.3% vs. 72.7% (p = 0.063), febrile neutropenia was 8.3% vs. 6.3% (p = 0.743), and thrombocytopenia was 48.7% vs. 51.5% (p = 0.813), in the FLOT and mDCF arms, respectively. Anemia was 59% in the FLOT arm and 100% in the mDCF arm (p < 0.001). However, grade 3–4 anemia was 7.7% in the FLOT arm and 24.2% in the mDCF arm (p = 0.052). It was observed that the rate of using primary GCSF prophylaxis was 84.6% in the FLOT arm and 90.9% in the mDCF arm (p = 0.421). The rate of using secondary GCSF prophylaxis was 5.1% in the FLOT arm and 18.2% in the mDCF arm (p = 0.079). The median duration of follow-up was 15.4 months in the mDCF arm and 9.4 months in the FLOT arm (p = 0.005). It was found that the treatment discontinuation rates were 8.1% vs. 3.0% (p = 0.361), and the rate of at least one dose reduction was 24.3% vs. 45.5% (p = 0.063) in the FLOT and mDCF arms, respectively. The delay of at least one dose was 66.7% in the mDCF arm and 32.4% in the FLOT arm (p = 0.004) (Table 6).

## 4. Discussion

In our study, it was revealed that the mPFS was significantly increased in the FLOT arm compared to the mDCF arm as a first-line treatment in patients with mG/mGEJ adenocarcinoma. The mOS’s in both FLOT and mDCF arms were similar. It was also found that the ORR was clinically meaningfully improved in FLOT arm compared to mDCF arm. Regarding hematological adverse events, anemia was statistically significantly less frequent, and hematological adverse events other than anemia were clinically meaningfully less frequent in the FLOT arm than those of in the mDCF arm.

Triplet regimens are the backbone of mGC. In the TAX-325 trial, it was reported that mOS was 9.2 months (95% CI, 8.4–10.6) and mPFS was 5.6 months (95% CI, 4.9–5.9) in the DCF arm [9]. In a phase 2 study in which DCF and ECF regimens were compared, it was found that mOS was 12.5 months (95% CI, 11.2–15.8) and mPFS was 7.5 months (95% CI, 6.2–9.7) in the DCF arm [18]. In a meta-analysis including 24 mDCF regimen studies, it was observed that mOS was 12.3 months (95% CI, 10.6–14.3) and mPFS was 7.2 months (95% CI, 5.9–8.8) in the pooled analysis [19]. In a phase II study with the FLOT regimen, it was reported mOS was 11.1 months and mPFS was 5.2 months [17]. In a study conducted with mFLOT regimen, mPFS was 4.4 months (95% CI, 2.9–5.9) [20]. In the AIO-FLOT3 study, which was a three-arm study, the B arm included limited metastatic gastric adenocarcinoma patients and the C arm included patients with extensive metastatic gastric adenocarcinoma. In the arm B, mOS was 15.9 months (95% CI, 7.1–22.9), and mPFS was 8.4 months (95% CI, 4.1–10.4) in patients not undergoing surgery. In the arm C, mOS was 10.7 months (95% CI, 9.1–12.8), and mPFS was 6.3 months (95% CI, 5.0–7.6) [16]. In our study, mPFS was 7.4 months (95% CI, 5.5–9.3) in the mDCF arm and 10.1 months (95% CI, 6.8–13.4) in the FLOT arm. The mPFS in the mDCF arm was similar to those of in the DCF pivotal study and mDCF studies [9,19]. The mPFS in FLOT arm was numerically higher than those of in previous FLOT studies [16, 17]. The contribution of number of metastatic sites <2 and ECOG PS of 0–1 on PFS was determined in both univariate and multivariate analyses. The positive effect of the FLOT regimen on the risk of progression in the multivariate analysis lost its significance. In our study, it was found that the mOS was 15.4 months (95% CI, 9.1–21.6) in the mDCF arm and 12.9 months (95% CI, 9.7–16.1) in the FLOT arm, and the difference was not statistically significant (p = 0.622). The mOS in the mDCF arm in our study was numerically higher than the mOS in the pivotal DCF study, the mOS in previous mDCF studies, and the mOS in prospective and retrospective DCF studies [9,18,21]. In our study, although the mPFS was significantly increased in the FLOT arm compared to the mDCF arm, it was thought that there might be some reasons why mOS’s in both arms were similar. In our study, the rate of the second-line chemotherapy was found to be significantly higher in the mDCF arm than in the FLOT arm. The rate of using combination chemotherapy as a second-line chemotherapy was significantly higher in the mDCF arm than that of in the FLOT arm. This difference in the use of the second-line chemotherapy might be shown as one of the reasons why the significant improvement in PFS did not remain as an advantage in OS. The widespread use of the concept of oligometastatic gastric cancer and local treatment methods might be shown as one of the reasons why patients with a single metastasis area had increased OS [22]. In our study, two patients in the mDCF arm underwent a metastasectomy. The overall survivals of these patients were over 30 months, and it is thought that it might contribute to prolonged OS in the mDCF arm.

In our study, it was found that the ORR in the FLOT arm (59%) was numerically lower than that of in arm B (66.7%), and numerically higher than that of in arm C (43.3%) of the AIO-FLOT3 study. Our study found that the ORR in the FLOT arm was numerically higher than that of in the pivotal DCF study (37%). In our study, the DCR in the FLOT arm (84.6%) and the DCR in arm B of the AIO-FLOT3 study (83.4%) were similar, but the DCR in our study was numerically higher than the DCR in arm C of the AIO-FLOT3 study (77.9%) [16]. The median duration of follow-up in our study was significantly higher in the mDCF arm than in the FLOT arm. One of the reasons of this discrepency migh be that the mDCF regimen is older than the FLOT regimen, and it was started earlier. In a phase II study conducted in Egypt, in which 72% of patients had metastatic disease, the ORR was %55.3 [23]. Our results were consistent with this phase II study.

High neuropenia rate in the pivotal DCF study directed us to perform GCSF prophylaxsis [24]. In our study, the application rates of primary GCSF and secondary GCSF prophylaxsis were similar in mDCF and FLOT arms. Anemia was significantly lower in the FLOT arm than that of in the mDCF arm. Other hematological adverse events were clinically significantly lower in the FLOT arm than that of in the mDCF arm. Grade 3–4 neutropenia was lower in our study (17.9%) than in arm B (46.3%) and arm C (42.1%) of the AIO-FLOT3 study. However, this study did not provide information about the application of GCSF prophylaxsis. Grade 3–4 thrombocytopenia and anemia in our study were higher than in the AIO-FLOT3 study. High GCSF application rates in our study might be count a possible explanation for that via a competitive microenvironment in bone marrow [16].

Our study has some limitations and strengths. Firstly, our study is a retrospective study with a limited number of patients. Secondly, adverse events data other than hematological adverse events could not be obtained completely. Nevertheless, it might be a guide in clinical practice in terms of presenting real-life data of detailed hematological toxicity profile comparing the FLOT and mDCF regimens.

In conclusion, it was revealed that mPFS was significantly increased in the FLOT arm compared to the mDCF arm in patients with mG/mGEJ adenocarcinoma as first-line treatment. The mOS’s in both FLOT and mDCF arms were similar. Favorurable hematological adverse events were obtained in the FLOT arm than those of in the mDCF arm. As far as we can reach, there is no randomized controlled prospective trial performed with FLOT regimen in metastatic GC and GEJC patients. In addition, to our best knowledge, this is the first retrospective study comparing the mDCF and the FLOT regimens as first-line treatment in mGC/mGEJC. The FLOT regimen might be considered an option as a first-line treatment in metastatic GC/GEJC patients with increased PFS and increased tolerability compared to the mDCF regimen. Further investigations of randomized controlled prospective trials with large patient groups are needed to provide a better knowledge on this issue.

## Figures and Tables

**Table 1:** Clinicopathologic characteristics of patients.

Variable	mDCF	FLOT	p-value
Number of patients, n (%)	33 (45.8)	39 (54.2)	-
Median age, years (IQR)	57 (50–62)	58 (47–66)	0.671
Elderly, n (%)			
≥65 years old	7 (21.2)	28 (71.8)	0.495
<65 years old	26 (78.8)	11 (28.2)	
Sex, n (%)			
Male	27 (81.8)	24 (61.5)	0.059
Female	6 (18.2)	15 (38.5)	
**Metastatic condition at initial diagnosis, n (%)**			
**Nonmetastatic**	**9 (27.3)**	**2 (5.1)**	**0.009**
**Metastatic**	**24 (72.7)**	**37 (94.7)**	
Metastatic site, n (%)			
Liver	14 (42.4)	17 (43.6)	0.921
**Peritoneum**	**22 (66.7)**	**15 (38.5)**	**0.017**
Bone	8 (24.2)	4 (10.3)	0.113
Others	18 (54.5)	19 (48.7)	0.622
No. of metastatic sites, n (%)			
<2	12 (36.4)	23 (59)	0.056
≥2	21 (63.6)	16 (41)	
Primary tumor localization, n (%)			
Cardia + EGJ	7 (21.2)	13 (33.3)	0.253
Fundus + Corpus	17 (51.5)	16 (41)	0.373
Antrum + Pylorus	9 (27.3)	10 (25.6)	0.876
Differentiation, n (%)			
Well + Moderate	7 (36.8)	9 (30)	0.619
Poor	12 (63.2)	21 (70)	0.619
Mucinous tumour, *n* (%)	10 (30.3)	17 (43.6)	0.246
ECOG PS, *n* (%)			
0–1	32 (97)	37 (94.9)	0.657
2	1 (3)	2 (5.1)	

**Table 2:** Univariate and multivariate cox regression models to estimate PFS.

Variable	Progression-free survival			Multivariate analysis		
	mPFS (months)	95% CI	Log-Rank	HR	95% CI	p-value
Elderly						
≥65 years old	7.8	5.3–10.2	0.742	-	-	-
<65 years old	9.8	5.3–14.4		-	-	-
Sex						
Male	7.8	5.5–10.0	0.278	-	-	-
Female	13.9	6.5–21.2		-	-	-
Metastatic site						
* **Liver**	**13.1**	**7.3**–**18.8**	**0.029**	0.55	0.30–10.1	0.057
** **Peritoneum**	**6.2**	**5.6**–**6.7**	**<0.001**	1.74	0.91–3.35	0.093
Bone	6.2	5.3–7.0	0.064	-	-	-
Others	7.9	5.9–9.8	0.210	-	-	-
**No. of metastatic sites**						
**<2**	**13.1**	**5.9-20.2**	**0.005**	**Ref**		
**≥2**	**6.6**	**5.3-7.9**		**1.93**	**1.00–3.72**	**0.048**
Primary tumor localization						
Cardia + EGJ	6.4	5.6–7.2	0.743	-	-	-
Fundus + Corpus	7.9	5.0-10.8		-	-	-
Antrum + Pylorus	9.8	8.3–11.2		-	-	-
Differentiation (n = 49)						
Well + Moderate	9.3	4.8–13.8	0.900	-	-	-
Poor	8.7	4.2–13.2		-	-	-
Mucinous tumour	6.4	3.3–9.4	0.740	-	-	-
**ECOG PS**						
**0-1**	**9.3**	**7.1**–**11.6**	**0.002**	**Ref**		
**2**	**3.3**	**0.6-6.0**		**10.38**	**2.15**–**49.9**	**0.004**
**First-line chemotherapy**						
**mDCF**	**7.4**	**5.5**–**9.3**	**0.041**	Ref		
**FLOT**	**10.1**	**6.8**–**13.4**		0.71	0.39–1.28	0.262

* The reference of this analysis is “no liver metastasis”.

** The reference of this analysis is “no peritoneal metastasis”.

**Table 3:** Univariate and multivariate cox regression models to estimate OS.

Variable	Overall survival			Multivariate analysis		
	mOS (months)	95% CI	Log-Rank	HR	95% CI	p-value
Elderly						
≥65 years old	12.3	0–26.9	0.333	-	-	-
<65 years old	14.9	12.6–17.2				
Sex						
Male	12.9	8.1–17.7	0.317	-	-	-
Female	15.4	11.9–18.8				
Metastatic site						
Liver	23.6	9.9–37.2	0.113	-	-	-
Peritoneum	10.9	4.6–17.2	0.294	-	-	-
* **Bone**	**10.7**	**9.3**–**12.0**	**0.005**	**2.56**	**1.22**–**5.35**	**0.012**
Others	13.3	9.9–16.7	0.194	-	-	-
**No. of metastatic sites**						
**<2**	**25.5**	**8.2**–**42.9**	**0.019**	Ref		
**≥2**	**12.3**	**9.2**–**15.5**		1.75	0.92–3.33	0.088
Primary tumor localization						
Cardia + EGJ	13.9	9.1–18.6		-	-	-
Fundus + Corpus	15.2	11.2–19.2	0.917	-	-	-
Antrum + Pylorus	14.9	9.9–20		-	-	-
Differentiation (n=49)						
Well + Moderate	19.3	8.0–30.7	0.968	-	-	-
Poor	13.9	10.9–16.9		-	-	-
Mucinous tumour (n = 27)	14.9	4.5–25.4	0.745	-	-	-
**ECOG PS**						
**0–1**	**15.2**	**12.5**–**17.8**	**<0.001**	**Ref**		
**2**	**5.0**	**0**–**10.6**		**8.78**	**2.44**–**31.5**	**0.001**
First-line chemotherapy						
mDCF	15.4	9.1–21.6	0.622	Ref		
FLOT	12.9	9.7–16.1		1.47	0.79–2.72	0.219

* The reference of this analysis is “no bone metastasis”.

**Table 4:** Second-line treatment exposure.

Variable, n (%)	mDCF	FLOT	p-value
Second-line treatment	23 (76.7)	7 (23.3)	<0.001
Combined chemotherapy regimen	21 (77.8)	6 (22.2)	<0.001
Single agent chemotherapy	2 (6.1)	1 (2.6)	0.437

**Table 5:** Best response rate analyses of patient with first-line treatment.

Parameter	mDCF	FLOT	p-value
Disease control rate, n (%)	24 (72.7)	33 (84.6)	0.216
Objective response rate, n (%)	16 (48.5)	23 (59)	0.373
Complete response, n (%)	2 (6.1)	2 (5.1)	0.863
Partial response, n (%)	14 (42.4)	21 (53.8)	0.334
Stable disease, n (%)	8 (24.2)	10 (25.6)	0.891
Progressive disease, n (%)	9 (27.3)	6 (15.4)	0.216
Median time to best response, months (min-max)	3.6 (0.69–9.8)	3.3 (1.6–14.7)	0.439

**Table 6:** Treatment exposure and hematologic advers events.

	mDCF		FLOT		*P* value	
Toxicities, n (%)	Grade 3–4	All	Grade 3–4	All	Grade 3–4	All
Neutropenia	6 (18.2)	24 (72.7)	7 (17.9)	20 (51.3)	0.980	0.063
**Anemia**	**8 (24.2)**	**33 (100)**	**3 (7.7)**	**23 (59)**	**0.052**	**<0.001**
Thrombocytopenia	2 (6.1)	17 (51.5)	1 (2.6)	19 (48.7)	0.459	0.813
Febrile neutropenia	2 (6.3)		3 (8.3)		0.743	
**Parameters**						
Primary GCSF prophylaxis, n (%)	30 (90.9)		33 (84.6)		0.421	
Secondary GCSF prophylaxis, n (%)	6 (18.2)		2 (5.1)		0.079	
**Median duration of follow-up, months (min-max)**	**15.4 (0.69**–**74.55)**		**9.4 (1.64**–**32.62)**		**0.005**	
Median duration of 1st line treatment, months (min-max)	7.43 (0.69–74.55)		7.46 (1.64–32.62)		0.928	
≥1 dose reduction, n (%)	15 (45.5)		9 (24.3)		0.063	
**≥1 dose delay (%), n (%)**	**22 (66.7)**		**12 (32.4)**		**0.004**	
Treatment cessation, n (%)	1 (3)		3 (8.1)		0.361	
